# DNA methylation dynamics in muscle development and disease

**DOI:** 10.3389/fnagi.2015.00019

**Published:** 2015-03-05

**Authors:** Elvira Carrió, Mònica Suelves

**Affiliations:** Institute of Predictive and Personalized Medicine of Cancer (IMPPC) and Health Sciences Research Institute Germans Trias I Pujol (IGTP)Badalona, Spain

**Keywords:** DNA methylation, muscle cell identity, exercise, aging, rhabdomyosarcoma, muscle pathologies

## Abstract

DNA methylation is an essential epigenetic modification for mammalian development and is crucial for the establishment and maintenance of cellular identity. Traditionally, DNA methylation has been considered as a permanent repressive epigenetic mark. However, the application of genome-wide approaches has allowed the analysis of DNA methylation in different genomic contexts revealing a more dynamic regulation than originally thought, since active DNA methylation and demethylation occur during cellular differentiation and tissue specification. Satellite cells are the primary stem cells in adult skeletal muscle and are responsible for postnatal muscle growth, hypertrophy, and muscle regeneration. This review outlines the published data regarding DNA methylation changes along the skeletal muscle program, in both physiological and pathological conditions, to better understand the epigenetic mechanisms that control myogenesis.

## Introduction

DNA methylation was the first discovered epigenetic modification (Holliday and Pugh, 1975; Riggs, 1975) being one of the best studied and most mechanistically understood. Chemically, it refers to the covalent addition of a methyl group (CH3) into the fifth position of the cytosine DNA nucleotide resulting in the modified base 5mC, frequently considered the fifth nucleotide, and well conserved among most plant, animal and fungal models (Feng et al., 2010). Cytosine methylation in mammals is almost restricted to the symmetrical CpG context (cytosine residue followed by a guanine bound by a phosphate union) (Ramsahoye et al., 2000), a dinucleotide globally underrepresented in the genome as a consequence of the spontaneous or enzymatic deamination of 5mC to thymine in the germ line (Bestor and Coxon, 1993). DNA methylation is critical for mammalian development being traditionally considered an heritable and stable silencing mark crucial for X-inactivation (Mohandas et al., 1981; Gartler and Riggs, 1983), genetic imprinting (Reik et al., 1987; Swain et al., 1987), silencing of genomic elements such as transposons to ensure genomic stability (Walsh et al., 1998; Gaudet et al., 2003), and maintenance of constitutively repressed centromeric and pericentromeric DNA satellite repeats (Gopalakrishnan et al., 2009). In addition, DNA methylation has an important role in gene regulation that depends on the genomic CpG context: promoter methylation is associated with gene silencing (Boyes and Bird, 1992; Hsieh, 1994), gene body methylation has variable effects on gene transcription (Gelfman et al., 2013; Yang et al., 2014), and intergenic methylation may affect gene expression through enhancer regulation (Stadler et al., 2011).

DNA methylation follows a bimodal distribution across the genome defined by the inverse correlation between 5mC and CpG density: CpG-poor DNA, which comprises most of the genome, shows high levels of 5mC, whereas high CpG-dense regions, termed CpG islands (Bird, 1986; Gardiner-Garden and Frommer, 1987) located mainly in the promoter regions of housekeeping and developmental genes, are largely resistant to DNA methylation (Takai and Jones, 2002; Illingworth and Bird, 2009). Notably, the surrounding regions to the CpG islands, named CpG island shores, are susceptible to be methylated in a tissue-specific manner correlating with changes in gene expression (Doi et al., 2009; Ji et al., 2010).

Strikingly, 20 years old seminal studies demonstrated that MyoD activation occurred in a demethylation-dependent manner (Brunk et al., 1996), establishing for the first time a direct link between DNA methylation and cellular differentiation. Since then, the importance of DNA methylation in modulating transcriptional programs during development and differentiation processes has been strongly supported by many studies. In the last few years, the development of large-scale sequencing strategies has made possible to achieve significant advances in understanding the regulatory role of DNA methylation at context-specific level and how the methylome affects cell identity. The aim of the present review is to discuss the major advances concerning the DNA methylation dynamics during myogenesis and its implications in muscle development and disease.

## Shaping the Cellular Identity Through DNA Methylation

**Figure 1:**
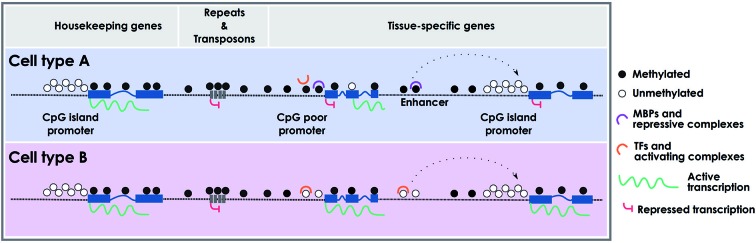
**Cell type-specific DNA methylation profiles**. Schematic representation of cell type-specific methylomes. CpG island promoters are usually protected from DNA methylation and are prone to active transcription. CpG-poor regions (intergenic) and repetitive elements are typically methylated, with the exception of enhancers and CpG-poor promoters that can be differentially methylated in a cell type-specific fashion. Intragenic regions can also be differentially methylated leading to specific cell-type transcripts. MBPs: methyl-binding proteins; TFs: transcription factors.

During cellular differentiation stem cells lose their plasticity and narrow their identity into particular differentiated cell types, which are usually stably maintained by epigenetic mechanisms. Embryonic stem cells (ESCs) are pluripotent cells showing very low methylation levels at CpG-rich sequences (Fouse et al., 2008), which along the differentiation processes into the three germ layers gain methylation in common but also specific regions (Meissner et al., 2008; Isagawa et al., 2011). Although ESCs completely lacking DNA methylation are viable and competent for self-renewal (Tsumura et al., 2006), they are partially blocked in the ability to initiate cellular differentiation (Jackson et al., 2004). Among others, the expression of mesodermal markers such as Brachyury, α-globin and βH1-globin are impaired or not maintained in embryo bodies (EBs) derived from ESCs lacking DNA methyltransferase activity (Jackson et al., 2004; Schmidt et al., 2012). Paradoxically, although there is a global gain of methylation during cellular differentiation, specific loci lose methylation in a cell-type dependent manner. While the gain of methylation mediates the silencing of pluripotency-associated and gamete-specific genes, as well as cell-type specific markers to avoid the expression of non-appropriate lineages, loss of methylation occurs in lineage-specific genes to define cellular identity (Nagae et al., 2011; Calvanese et al., 2012; Nazor et al., 2012; Figure 1). Interestingly, these demethylated sequences are not only restricted to promoter regions, but are also found in distal gene sequences and intronic regions, which might affect enhancer elements, alternative promoters and alternative splicing variants in a cell-type dependent manner (Meissner et al., 2008; Stadler et al., 2011).

### Genome-wide Studies Addressing the Skeletal Muscle Methylome

Several studies comparing DNA methylation signatures between cell types and tissues indentified muscle-specific differentially methylated regions. As mentioned before, CpG islands are found at over half of all human gene promoters and are often free of methylation (Ioshikhes and Zhang, 2000; Lander et al., 2001; Saxonov et al., 2006). The analysis of 17,000 CpG islands in five human samples (blood, sperm, brain, skeletal muscle and spleen) showed a common set of methylated CpG islands in all somatic samples, associated with genes that are essential for development, neurogenesis, and segment specification (Illingworth et al., 2008). In addition, this study identified 178 CpG islands specifically hypermethylated in muscle tissue, showing the skeletal muscle the highest percentage of methylated CpG islands (8.3%) (Illingworth et al., 2008). Sorensen et al. analyzed the methylation level of 200 lineage-specific differentiation markers in muscle-progenitor cells (MPCs), adipose stem cells (ASCs) and bone marrow mesenchymal stem cells (BMMSCs), using Methyl-DNA Immunoprecipitation (MeDIP) followed by microarray hybridization. The results showed that despite most of the lineage-specific promoters did not show changes in DNA methylation, important lineage-specific markers were hypermethylated in MPCs, correlating with gene silencing, such as GATA-2, AQP7 and ACACA (adipogenic lineage), RUNX2 and THY1 (osteogenic lineage), INS2 and PDX1 (pancreatic development), KRT1A (skin development) and NEFH (neurogenic lineage) (Sorensen et al., 2010). Later on, Fernandez et al. analyzed 1,505 CpGs surveying 808 gene promoters in 1,628 human samples including 22 muscle tissues. This study identified 183 muscle-specific differentially methylated CpGs (dmCpG) on gene promoters including GLI2, CREBBP, PDGFRA, CD34 and CARD15 genes (Fernandez et al., 2012). In parallel, using the methylation array Illumina Infinium 27K DNA Methylation BeadChIP, 47 genes were found specifically hypomethylated in muscle tissue, including genes encoding for proteins located in contractile fibers such as Obscurin, Myotilin, and Myh7 (Calvanese et al., 2012). At the same time, a genome-wide DNA methylation analysis was performed to compare 205 human pluripotent stem cells and 130 somatic samples, including skeletal muscle, by using the complete Illumina Infinium 450K DNA Methylation BeadChIP array. The results reveled 782 and 621 unique CpGs exclusively hypomethylated and hypermethylated, respectively, in skeletal muscle samples, being the hypomethylated regions functionally enriched in response to peptide hormone stimulus and wound healing (Nazor et al., 2012).

All these studies highlighted a specific DNA methylation signature of skeletal muscle cells compared to other cell-types, but did not address when this specific pattern was acquired. A recent study comparing human proliferating myoblasts (MBs) and differentiated myotubes (MTs), using the non-promoter-oriented RRBS method, showed no significant methylation changes during myogenic terminal differentiation (Tsumagari et al., 2013b). In this study the authors also compared non-muscle cell cultures with myoblast/myotube cultures and found similar numbers of differentially hyper- and hypo-methylated sites (9,592 and 10,048, respectively). The regions specifically hypermethylated at MB/MT cells were strongly associated with genes encoding for transcription factors, especially homeobox and T-box genes, while muscle hypomethylation was observed at contractile fiber genes. The myogenic hypermethylation found in specific subregions of all four HOX gene clusters pointed out the involvement of DNA methylation (at 5’ and internal promoters, as well as at intragenic and intergenic enhancers) in the fine-tune HOX genes regulation during development (Tsumagari et al., 2013a). In addition, the comparison of skeletal muscle methylome vs. other tissues showed that the 94% of differentially methylated sites were hypomethylated in muscle, and 47% of them were also hypomethylated in MB/MT cells, suggesting that DNA demethylation occurs gradually along the myogenic process (Tsumagari et al., 2013b). Very recently, Miyata et al. addressed the chronological alterations in DNA methylation during myogenic differentiation comparing human myoblast and myotube cultures using the Illumina Infinium 450K DNA Methylation BeadChIP array. Interestingly, they found hypermethylation in the binding sites for the transcription factor ID4 and ZNF238 during the myogenic progression, contributing to myotube formation (Miyata et al., 2014). Intriguingly, they also observed a small but global increase in DNA methylation levels during terminal differentiation occurring at genes involved in muscle contraction and muscle system process.

In summary, all these studies comparing methylation profiles between cell-types show both common and specific features among all of them. A general consensus about the importance of muscle-specific demethylation during lineage specification is prevalent among studies, and remarkable, identity-dependent methylation changes occur mostly during early cell fate decisions while fewer DNA modifications take place later during terminal cellular maturation.

## DNA Demethylation-dependent Activation of Muscle-specific Genes

Seminal works of Helen Blau in the early eighties showed that the fusion of human non-muscle cells with mouse muscle cells resulted in heterokaryons expressing human muscle-specific genes, under conditions in which there was no DNA replication (Blau et al., 1983, 1985). In addition, these pioneer experiments showed that HeLa cells treated with 5-azacytidine prior to fusion also formed heterokaryons, which expressed muscle-specific genes suggesting that DNA demethylation could be involved in muscle gene activation (Chiu and Blau, 1985). Importantly, a decade later it was shown that *MyoD* activation occurred in a demethylation-dependent manner (Brunk et al., 1996), and H. Blau’s laboratory demonstrated active demethylation of human MyoD in fused non-dividing heterokaryons (Zhang et al., 2007). Several examples in different cellular models have grounded the hypothesis that lineage-specific demethylation in tissue-specific progenitors is required for cellular differentiation, since DNA demethylation generates a transcriptionally permissive chromatin. We present below some examples of DNA demethylated myogenic genes.

### 5-azacytidine Triggers and Enhances Muscle Differentiation

As previously mentioned, the finding of the *MyoD* tissue-specific demethylation was the seminal work that linked for the first time DNA methylation and cell fate commitment. In 1973, Peter Jones was investigating the effect of 5-azacytidine, a new chemotherapeutic drug, in mouse embryonic fibroblasts (10T1/2 cells) when he realized that treated cells turned into a huge syncytium of multinucleated cells (Constantinides et al., 1977). After confirming the myogenic phenotype of 10T1/2 treated cells and demonstrating that the tested drug was a potent inhibitor of DNA methylation (Jones and Taylor, 1980), *MyoD* was identified as the key gene involved in muscle reprogramming (Lassar et al., 1986; Davis et al., 1987). In addition, the same myoblastic conversion occurred in 10T1/2 fibroblasts transfected with an antisense cDNA against the maintenance DNA methyltransferase DNMT1 (Szyf et al., 1992), suggesting the involvement of DNA methylation in *MyoD* gene regulation. Analysis of treated 10T1/2 fibroblasts revealed the highly methylated state of the CpG island surrounding the *MyoD* promoter, however this CpG island was constitutively free of methylation in mouse analyzed tissues (Jones et al., 1990) raising the question whether the demethylation of *MyoD* promoter was indeed the signal required to initiate the myogenic program. The answer came when Brunk et al. showed the specific demethylation of a distal regulatory region located at—20 Kb of *MyoD* TSS during somitogenesis (Brunk et al., 1996). They observed by sodium bisulphite conversion that three CpGs located in this enhancer region were partially methylated in liver, heart, and brain mouse tissues (50–60%), and in fibroblast and neuroblastoma cells (50–80%), while they were almost totally unmethylated in forelimb or hindlimb muscles (17%) and in C2C12 myoblast cell line (8%). Moreover, they analyzed the methylation state during somitogenesis observing that the distal enhancer was heavily methylated in the presomitic mesoderm and became almost completely demethylated between somites 6 and 10 as myogenesis progressed. Intriguingly, mutations of these dmCpG did not cause precocious activation of *MyoD* in trangenic mice, which implied that DNA methylation was not sufficient for gene inactivation. Moreover, the authors showed that demethylation of *MyoD* distal enhancer occured prior to gene activation, although *MyoD* expression did not immediately follow enhancer demethylation, suggesting that active demethylation was required but was not sufficient for *MyoD* activation (Brunk et al., 1996). In parallel and intriguingly, Takagi et al. observed an enhancement in myotube formation upon overexpression of DNMT1 in myoblast C2C12 cell line (Takagi et al., 1995). Using methods based in methylation sensitive enzymes they detected a positive correlation between *MyoD* expression and exon 1-2 hypermethylation, which would be in agreement with the idea that DNA methylation in gene bodies correlates with gene expression.

Subsequent studies also observed an enhancement of muscle differentiation in C2C12 MBs treated with 5-azacytidine, resulting in higher myotube formation with enhanced maturity (Hupkes et al., 2011). A recent study analyzed the effect of 5-azacytidine treatment on C2C12 cell cycle regulation. The results showed that inhibition of DNA methylation increased the expression of checkpoint genes involved in cell cycle progression, arrested cell cycle, and up-regulated myogenic transcription factors enhancing myogenesis, although no analysis of methylation state of myogenic genes was performed (Montesano et al., 2013).

### Myogenin DNA Demethylation During Myoblast Differentiation

The correlation between muscle differentiation and DNA demethylation was further underscored by the finding that *Myogenin* promoter became demethylated at the onset of C2C12 muscle differentiation. Lucarelli et al. reported that the methylation status of a single CpG site at 340 bp upstream from the TSS affected *Myogenin* transcription in mouse tissues and C2C12 cells. *Myogenin* expression in differentiated muscle cells correlated with lack of methylation, while methylated non-muscle tissue (spleen and brain) and proliferating MBs showed no gene expression (Lucarelli et al., 2001). In addition, the same lab showed that proliferating MBs treated with 5′-Aza-2′-deoxycytydine increased *Myogenin* expression due to a reduction in DNA methylation, highlighting the regulatory role of DNA methylation in *Myogenin* expression (Scarpa et al., 1996). Later on, Palacios et al. confirmed these results and addressed *Myogenin* promoter DNA methylation dynamics during somitogenesis showing anterior-posterior DNA demethylation correlatives to *Myogenin* expression and muscle development (Palacios et al., 2010). Mechanistically, they showed that efficient activation of *Myogenin* promoter required DNA demethylation following binding of SIX1 and MEF2 proteins to favor the competition between these two transcription factors and methylated DNA binding proteins. In this regard, Oikawa et al. demonstrated that the methyl-CpG-binding protein CIBZ suppressed myogenic differentiation by direct binding to the methylated *Myogenin* promoter, and importantly DNA demethylation prevented the binding of CIBZ repressor allowing the binding of MyoD/Pbx/Meis activator complex (Oikawa et al., 2011). Finally, Strom’s laboratory also reported DNA demethylation in the *Myogenin* promoter but at non-CpG dinucleotides, claiming that demethylation occurs more rapidly at non-CpG than at CpG dinucleotides (Fuso et al., 2010). Although most studies restrict non-CpG methylation to ESCs and brain tissue (Ramsahoye et al., 2000; Lister et al., 2009; Guo et al., 2014), it has been estimated that 7% of cytosines within the sequence CCAGG or CCTGG are methylated in human skeletal muscle (Barres et al., 2009). In the near future it would be important to address the role of non-CpG methylation in muscle development.

### Other Genes Becoming DNA Demethylated During Myoblast Cell Differentiation

Recently, other studies have also reported changes in DNA methylation affecting the activation of muscle-related genes. This is the case of *Desmin* gene, which contains a CpG island covering the TSS and a proximal enhancer extensively studied at the epigenetic level in MBs, MTs and peripheral blood, as non-muscle control sample. While the CpG island is fully unmethylated in all samples, the enhancer shows lack of methylation exclusively in myogenic cells, both in MBs and MTs, although unfused MBs exhibit lower level of *Desmin* expression (Lindahl Allen et al., 2009). The study of the histone modifications revealed that MTs showed increase positive /open chromatin marks (H3K4me3 and H3K4me2) compared to MBs, suggesting that DNA demethylation may provide a transcriptionally poised state that would be activated during differentiation, upon the acquisition of transcription factors and positive histone marks.

The transcription factor SIX1 is essential for the formation of multiple organs including the skeletal muscle (Grifone et al., 2005). Wu et al. reported that SIX1 was exclusively expressed in porcine adult skeletal muscle and displayed the highest expression levels in fast muscles (Wu et al., 2011). The comparison of DNA methylation levels of SIX1 core promoter in several tissues revealed that skeletal muscle showed the lowest levels correlating with the higher gene expression, and similarly different methylation status were also observed in different skeletal muscles consistent with the reported variations in expression levels (Wu et al., 2013).

Expression of α-smooth muscle actin (α-SMA) is a key indicator of myofibroblast differentiation into fibroblast, and its accumulation in tissue remodeling and fibrosis leads to a deleterious excess of extracellular matrix components (Hinz et al., 2003; Hinz, 2007; Klingberg et al., 2013). α-SMA gene contains three CpG islands and a differential methylated state was reported in the second and third islands in α-SMA expressing myofibroblast vs. non-expressing epithelial cells (Hu et al., 2010). In addition, inhibition of DNA methyltransferase activity led to significant induction of α-SMA expression, while ectopic expression of DNMTs suppressed its expression (Hu et al., 2010). Interestingly, all these data suggest that the enhancement of DNA methylation, by DNMT activity, could be a key mechanism for reducing myofibroblast differentiation.

### The Role of TET Proteins in Active Demethylation in Muscle Differentiation

The recent demonstration that Ten-eleven translocation family of protein dioxygenases (TET1-3) had the capacity to convert 5mC into 5-hydroxymethylcytosine (5hmC) raised the possibility that 5hmC might constitute a distinct epigenetic state contributing to dynamic changes in DNA methylation (Tahiliani et al., 2009; Ito et al., 2010). 5hmC was found in many tissues and cell types, although with diverse levels of abundance being enriched in ESCs and certain types of neurons (Kriaucionis and Heintz, 2009; Ito et al., 2010; Szwagierczak et al., 2010). Recently, an ultra-sensitive and accurate isotope based HPLC-MS method was used to precisely determine the levels of h5mC in different mouse tissues revealing that skeletal muscle showed an intermediate level estimated between 0.15–0.17% of cytosine residues (Globisch et al., 2010).

As mentioned above, dynamic changes in DNA methylation occur during early development (Surani et al., 2007; Hajkova et al., 2010) and DNA demethylation takes place in a highly locus-specific fashion upon development (Ma et al., 2009; Wu and Zhang, 2010). Base-resolution analysis of 5hmC in ESCs revealed high levels of 5hmC (and reciprocally low levels of 5mC) at regions of low CpG content and near, but not on, transcription factor binding sites, suggesting that TET proteins might influence gene expression (Yu et al., 2012). TET1^−/−^ mice were viable and fertile but about 75% of the homozygous mutant pups had smaller body size (Dawlaty et al., 2011). Gene expression array analysis showed 221 significantly deregulated genes, including developmental skeletal muscle and muscle contraction genes (Dawlaty et al., 2011). The very recent generation of TET1/2/3 (TKO) ESCs showed EBs totally depleted in 5hmC with a concomitant increase in global 5mC levels (Dawlaty et al., 2014). Moreover, TKO EBs expressed reduced levels of mesodermal and endodermal markers. Furthermore, global gene expression and DNA methylation analyses of TKO EBs revealed promoter hypermethylation and deregulation of genes implicated in embryonic development and differentiation, including skeletal muscle development genes being one of the top ten GO categories (Dawlaty et al., 2014). All these observations suggest that TET enzymes are critical during ESC differentiation by regulating promoter methylation levels of a subset of developmental regulators and lineage commitment genes, and thus enabling their activation by differentiation-induced and lineage-specific demethylation.

Very interestingly, it was reported that NOTCH1 receptor and its ligand DLL1 showed high levels of 5hmC in myogenic differentially methylated regions in skeletal muscle, cerebellum and heart (Terragni et al., 2014). Notch signaling is involved in the regeneration of injured skeletal muscle, brain function and cardiac disease, and these results suggest that hypomethylation and/or hydroxymethylation may help to control gene expression in a tissue and stage-specific manner (Terragni et al., 2014).

It has been widely reported that enhancer activity is modulated through histone modifications, histone variant deposition and nucleosome stability. Interestingly, during neuronal differentiation of P19 cells and adipogenic differentiation of 3T3-L1 cells DNA hydroxymethylation was an early event of enhancer activation (Serandour et al., 2012). Recently, a compendium of regulatory elements driving muscle differentiation was identified based on chromatin signatures and MyoD recruitment (Blum et al., 2012). In the near future, it would be worth to analyze whether the acquisition of 5hmC in myogenic-specific distal regulatory regions might activate enhancer elements leading to the expression of muscle-differentiation genes.

## DNA Methylation Changes During the Skeletal Muscle Aging

Aging is accompanied by a progressive decline in adult tissue-specific stem cells functions, resulting in less effective tissue homeostasis and repair. Aging-associated mechanisms are not yet totally understood, but DNA methylation changes and other epigenetic mechanisms contribute to aging phenotypes. Early studies measuring global DNA methylation, using biochemical methods, showed a progressive depletion of 5-methylcytosine in senescent normal fibroblast (Wilson and Jones, 1983), and similar changes were also observed in aging mouse tissues and human cells (Wilson et al., 1987). Comprehensive DNA methylation analyses have been facilitated by the application of genome-wide technologies revealing numerous methylation alterations, both hyper- and hypomethylation changes, occurring in several tissues during aging (Grönniger et al., 2010; Maegawa et al., 2010; Rakyan et al., 2010; Bocker et al., 2011; Hernandez et al., 2011; Bell et al., 2012; Heyn et al., 2012; Beerman et al., 2013). Although the functional consequences of this age-related methylation drift remain unknown, the alterations in DNA methylation state would create epigenetic mosaic between aging-stem cells affecting their proliferative and differentiation capacities (Issa, 2014).

A recent genome-wide study comparing DNA methylation patterns in postmitotic skeletal muscle taken from healthy young (18–27 years of age) and old (68–89 years of age) males showed a predominant pattern of hypermethylation in the DNA of aging skeletal muscle (Zykovich et al., 2014). Most of the methylation changes occurred intragenically, being underrepresented in promoter regions. Using the Illumina Infinium 450K DNA Methylation BeadChIP array 2,114 genes were identified with at least one dmCpG site located intragenically. The Tubulin-folding cofactor D (TBCD), involved in microtubule functions, was the highest hypermethylated gene in aged-samples. Gene ontology analysis of genes with two or more intragenic dmCpG site showed that the most enriched terms and pathways were “muscle cell” (*P* = 0.0004) and “axon guidance signaling” (*P* = 6.17E–10), suggesting that the dmCpG sites identified within the aged group were relevant to muscle tissue functions and neuromuscular junctions (Zykovich et al., 2014). In fact, one of the major contributors in sarcopenia is the motor unit loss or denervation, and these results point out that the process might be affected by DNA methylation. Notably, NFATC1 gene involved both in the regulation of signaling at the neuromuscular junction, and acting as transcriptional regulator of muscle fibers in direct response to electrical stimulation via calcium/calmodulin signaling (McCullagh et al., 2004; Rana et al., 2008; Salanova et al., 2011) was hypermethylated in aged-skeletal muscles. Interestingly, DNA methylation changes on NFATC1 and other genes within the axon guidance signaling pathway may alter transcriptional events leading to the loss of proper reinnervation at the neuromuscular junction during muscle turnover resulting in muscle wasting.

Very recently, Jin et al. analyzed genome-wide DNA methylation changes in skeletal muscle between young and middle-aged pigs, as a good biomedical model for human studies, using a methylated DNA immuprecipitation sequencing approach. Contrarily to the previous study, they found a global tendency towards loss of DNA methylation in middle-aged pigs compared to the young group, enriched also in gene-body regions (Jin et al., 2014). The intersection of genes that presented DMRs in their promoters and gene body with the 288 genes potentially involved in the human aging process (according to the Human Aging Genomic Resources database) revealed 12 known age-related genes including FoxO3 and FGFR1. FoxO3 is an essential transcription factor involved in lysosomal proteolysis in muscle, by activating autophagy and proteosomal pathways (Mammucari et al., 2007; Zhao et al., 2007). Interestingly, FoxO3 becomes upregulated in aged muscles correlating with lower gene-body methylation status (Jin et al., 2014). Contrary, FGFR1 shows an opposite function to that of FoxO3, inhibiting the atrophy of skeletal muscle (Eash et al., 2007), and it is found downregulated and hypermethylated in gene-body in middle-aged pigs. These results highlight the participation of DNA methylation changes enhancing proteolysis and protein catabolic processes during aging, which suggest an important role of epigenetic mechanisms in aged-related muscle-atrophy.

A potential issue regarding these two genome-wide skeletal muscle studies is the use of muscle biopsies that include several non-muscle cell types, which might confound to some point muscle-specific methylation signatures. In the future, it will be necessary to evaluate whether DNA methylation differences between young and aged/sarcopenic satellite cells may affect muscle stem cell function. Notably, *MyoD* hypermethylation was reported in aged blood and brain samples (Hernandez et al., 2011; Fernandez et al., 2012) raising the question if it could be also differentially methylated in aged satellite cells. Finally, the identification of age-related DNA methylation patterns was used to build a model of biological aging (Hannum et al., 2013). Interestingly, some of the differentially methylated probes distinguishing young from old skeletal muscles were included in these age-predicting markers (Zykovich et al., 2014). In this regard, the ability to predict biological age from DNA methylation markers could be envisioned as a health assessment to prevent and diagnose aged-related diseases.

## DNA Methylation Alterations in Rhabdomyosarcoma and Muscle Diseases

Epigenetic modifications in concert with genetic mechanisms regulate transcriptional activity in normal tissue but are often dysregulated in diseases. Here we discuss recent progress regarding DNA methylation alterations in muscle-related pathologies.

### Rhabdomyosarcoma Tumorigenesis

Epigenetic aberrations have been well established in cancer: the genome of cancer cells is globally hypomethylated compared to normal tissues, but at the same time CpG islands in regulatory regions of tumor suppressor genes are often hypermethylated, being both events important for the origin and progression of many human cancer (Berdasco and Esteller, 2010). Rhabdomyosarcoma (RMS) is the most common soft-tissue sarcoma of childhood, with skeletal muscle presumed origin because of its myogenic phenotype. However, unlike normal MBs RMS cells differentiate poorly both *in vivo* and in culture (Keller and Guttridge, 2013). Experiments in the late eighties showed that the treatment of the RMS cell line RMZ-RC2 with the demethylating agent 5-azacytidine resulted in an increased differentiation capacity, suggesting that aberrant DNA methylation was repressing differentiation genes in this RMS cell line (Lollini et al., 1989). In addition, the comparison of DNMTs levels between normal skeletal muscles and RMS tumors showed significant higher expression of DNMTs in both alveolar and embryonal RMS subtypes, which have distinct etiologic and clinical behaviors (Chen et al., 1998). Candidate gene approaches identified DNA methylation changes in RMS tumors in *MyoD* (Chen et al., 1998), *p21WAF1* (Chen et al., 2000), *RASSF1* (Harada et al., 2002), *PAX3* (Kurmasheva et al., 2005), *plakoglobin* (Gastaldi et al., 2006), *FGFR1* (Goldstein et al., 2007), *JDP2* (MacQuarrie et al., 2013), *BMP2* (Wolf et al., 2014), and *CAV1* (Huertas-Martínez et al., 2014) genes. Recently, a genome-wide analysis of promoter CpG island methylation between RMS subtypes and skeletal muscles revealed RMS-specific hypermethylation in genes associated with tissue development, differentiation and oncogenesis such as *DNAJA4*, *HES5*, *IRX1*, *BMP8A*, *GATA4*, *GATA6*, *ALX3* and *P4HTM*, implicating aberrant DNA methylation in the pathogenesis of RMS (Mahoney et al., 2012). In addition, cluster analysis showed that embryonal and alveolar subtypes had distinct methylation patterns, with the alveolar subtype being enriched in DNA hypermethylation of polycomb target genes (Mahoney et al., 2012). Importantly, the different DNA methylation signatures between RMS subtypes might aid to define tumor subtype, clinical prognosis and treatment response of RMS tumors.

The connection between miRNAs, tissue differentiation and malignant transformation emerged long time ago (Reinhart et al., 2000; Calin et al., 2002). Although some miRNAs can act as oncogenes, miRNAs identified as de-regulated in cancer are more commonly tumor supressors, that are down-regulated by several mechanisms including epigenetic silencing (Rota et al., 2011). In the last few years, the list of miRNAs undergoing promoter hypermethylation has been rapidly expanded in many human tumors, highlighting the transcriptional repression of miRNAs by DNA methylation as a common feature in human cancer (Lopez-Serra and Esteller, 2012). Recent evidences have shown low expression of miR-206 (Missiaglia et al., 2010), miR-1 and miR-133a (Rao et al., 2010), and miR-203 (Diao et al., 2014) in RMS cells which have been correlated with higher proliferation rates, impaired differentiation and poor overall survival. Notably, it was recently demonstrated that miR-203 was frequently down-regulated in both RMS cell lines and RMS biopsies by promoter hypermethylation, and importantly, it can be reactivated by DNA-demethylating agents inhibiting tumor growth and migration capacity, and promoting terminal differentiation (Diao et al., 2014). All these data allow envisioning the use of combined treatment including epigenetic drugs for the treatment of the aggressive RMS tumors.

### Epigenetic Determinants in Other Muscle Pathologies

Most muscle pathologies are caused by gene mutations; however, for some recessive myopathies a number of affected individuals show only monoallelic mutations, pointing out that epigenetic mechanisms could be affecting the expression of the second allele. Recessive core myopathies, where the skeletal muscle Ryanodine receptor gene (*RYR1*) is mutated (Quane et al., 1993; Jungbluth et al., 2005), and dysferlinopathies, caused by mutations in Dysferlin (*DYSF*) gene (Liu et al., 1998), show a percentage of patients with only an identified mutated allele. Importantly, for core myopathies it was shown that the treatment of cultured patient skeletal-muscle MBs with 5-Aza-2′-deoxycytidine reactivated the expression of the non-mutated allele, suggesting the implication of DNA hypermethylation in the silencing of the gene (Zhou et al., 2006). However, detailed bisulphite sequencing analysis of all CpG islands located in *RYR1* and *DYSF* genes showed no differences in DNA methylation levels between healthy and affected individuals (Zhou et al., 2006; Gallardo et al., 2014). These results raised the question of which would be the epigenetic mechanism responsible for the reported RYR1 monoallelic expression. The most plausible hypothesis according to the authors was a genomic-imprinting mechanism mediated by differentially methylated imprinting control regions, since they observed an association with the sex of the non-transmitting parent, and a tissue-specific nature of the epigenetic silencing (Zhou et al., 2006). Although other regulatory mechanisms cannot be definitively excluded, the results suggesting an altered epigenetic regulation involved in these muscle pathologies support the use of epigenetic drugs to reverse or to ameliorate the symptons of the disease.

Juvenile dermatomyositis (DM) is a severe chronic childhood autoimmune disease showing the affected children muscle weakness caused by chronic muscle damage (Christen-Zaech et al., 2008; Feldman et al., 2008). Comparison of genome-wide DNA methylation profiling, by Illumina Infinium Human Methylation27K BeadChip array, revealed 27 genes with significant methylation differences between normal and juvenile DM diagnosed muscle biopsies (Wang et al., 2012). Although previous gene expression studies showed alterations in the expression of genes involved in immune response, vascular remodeling and endoplasmic reticulum response to acid stress (Nagaraju et al., 2005; Chen et al., 2008), no significant methylation alterations in those genes were found in juvenile DM. However, among the 27 differentially methylated genes several HOX genes (HOXC11, HOXD3 and HOXD4) and the developmental transcription factor WT1 were found hypomethylated in juvenile DM samples, as well as in other types of idiopathic inflammatory myopathies (IIMs) with muscle weakness, such as juvenile polymyositis (Wang et al., 2012). The similar methylation alterations of WT1 and homeobox genes found in IIMs provided additional evidences that the damaged muscles in these children had self-renewal capacity, stimulating the muscle stem cell pool towards muscle repair. Interestingly, these results suggest that the homeobox and WT1 genes are epigenetically marked to facilitate the repair process in response to the muscle damaged occurring during disease pathogenesis.

## Effects of Exercise on Skeletal Muscle DNA Methylation Signature

There is no doubt that physical exercise contributes to improve human health. Indeed, a large number of studies showed that exercise alters the expression of genes that affect glucose and lipid metabolism, mitochondrial function, as well as, transcription factors, myogenic regulatory factors and myokines (Pilegaard et al., 2000, 2003; Coffey and Hawley, 2007; Egan et al., 2010). Although the global effect of exercise on DNA methylation is still not well known, some studies have address whether DNA methylation plays a role in exercise-induced gene expression.

Barrès et al. analyzed DNA methylation levels of the *vastus lateralis* skeletal muscle from young and healthy men and women before and after 20 min of acute exercise. Luminometric methylation assay (LUMA) showed a small but very rapid global DNA methylation decrease in young muscles (Barrès et al., 2012). Although factors involved in expression of muscle-specific genes such as MyoD did not show methylation differences, several metabolic gene promoters such as PGC-1α, TFAM, PPARγ, PDK4 and citrate synthase showed lower methylation, by MeDIP-PCR analysis, correlating with higher expression levels after exercise. Similarly, promoter methylation of PGC-1α, PPARγ, and PDK4 was decreased in mouse *soleus* muscles 45 min after *ex vivo* contraction correlating with increased gene expression (Barrès et al., 2012). In parallel, another study using MeDIP-ChIP analysis evaluated the effect of 6 months of moderate aerobic exercise, in persons with and without diabetes type 2 family histories. The results showed DNA methylation changes in 134 genes after 6 month long training period, independently of the familiar history (Nitert et al., 2012). Most of the genes showed decreased methylation (115 out of 134) and were involved in retinol metabolism, calcium-signaling pathway, and starch and sucrose metabolism. Among them, the transcription factors RUNX1 and MEF2A, both important for muscle physiology, increased their expression after moderate exercise correlating negatively with DNA methylation changes (Nitert et al., 2012).

Altogether, these results suggest that physical exercise may modify DNA methylation patterns, although future studies will be needed to deeper understand the involvement of epigenetic mechanisms in the beneficial effects of regular exercise on human health.

## Conclusions

**Figure 2:**
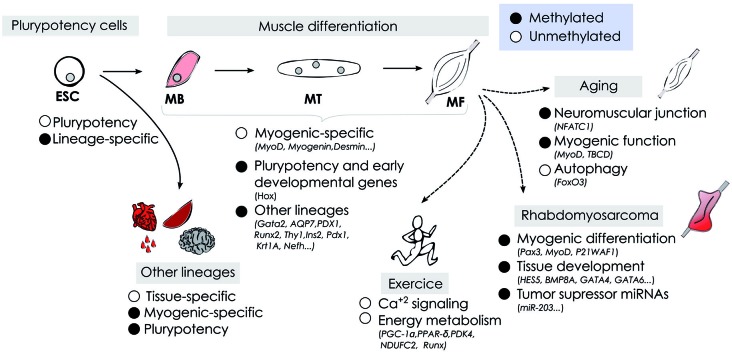
**Schematic representation of DNA methylation dynamics during myogenesis under physiological and pathological conditions**.

DNA methylation plays an important role in mammalian development, as illustrated during skeletal muscle cell fate commitment and differentiation. During development muscle stem cells acquire an unique DNA methylation signature associated with its specialized functions, and specific-myogenic factors are activated in a demethylation-dependent manner. DNA methylation patterns are not fixed but dynamic, and can be modulated by external influences. We have highlighted the recent data regarding how skeletal muscle methylome changes in response to physical exercise, aging and in muscle-related pathologies including cancer (summarized in Figure 2). The development of whole-genome approaches has contributed with important advances in understanding the regulatory role of DNA methylation at context-specific level and how the methylome affects cell identity. However, many important questions remain open regarding the demethylation mechanisms involved in specific-myogenic demethylation, the function of non-CpG methylation in muscle cells, and the role of 5-hydroxymethylation modulating proximal and distal regulatory regions affecting gene expression. In addition, the intense and recent interest in cellular reprogramming in the field of regenerative medicine emphasizes the importance of identifying cell type-specific epigenetic signatures to ensure a safety reprogramming in stem cell-based therapies (Barrero et al., 2010). Finally, the recognition of DNA methylation as a significant contributor to normal muscle physiology and its alterations in pathological processes, as well as in aging open new avenues to envision a near future where epigenetic therapies will be included in the treatment of muscle-related diseases.

## Author Contributions

EC and MS wrote the manuscript. Both authors read and approved the final manuscript.

## Conflict of interest statement

The authors declare that the research was conducted in the absence of any commercial or financial relationships that could be construed as a potential conflict of interest.

## Abbreviations

ChIP, Chromatin inmunoprecipitation; DMRs, Differentially methylated regions; DNMT, DNA methyltransferase; EB, Embryo body; ESCs, Embryonic stem cells; MB, Myoblast; MT, Myotube; MeDIP, Methyl DNA immunoprecipitation; MPC, muscle progenitor cell; RMS, Rhabdomyosarcoma; RRBS, Representative reduced bisulphite sequencing; TET, Ten-eleven translocation; TSS, Transcription start site; 5C, 5-cytosine; 5mC, 5-methylcytosine; 5hmC, 5-hydroxymethylcytosine.
